# PROFILING DEMENTIA FAMILY CAREGIVING NETWORK MEMBERSHIP AND PSYCHOSOCIAL FUNCTIONING

**DOI:** 10.1093/geroni/igae098.2419

**Published:** 2024-12-31

**Authors:** Amanda Leggett, Srabani Haldar, Wenhua Lai, Sophia Tsuker, Natasha Nemmers

**Affiliations:** Wayne State University, Detroit, Michigan, United States; Wayne State University, Detroit, Michigan, United States; Wayne State University, Detroit, Michigan, United States; Wayne State University, Detroit, Michigan, United States; Wayne State University, Detroit, Michigan, United States

## Abstract

Family caregiving research predominantly takes a “primary” caregiver lens, while ignoring the multifaceted caregiving networks surrounding a person living with dementia (PLWD). We explore profiles of dementia family caregiving networks based on their compositional and psychosocial characteristics. Our sample includes 184 dementia family caregiving networks drawing data from the 2017 National Health and Aging Trends Study and the associated National Study of Caregiving. Latent profile analysis was run to define distinct network profiles considering compositional (e.g., proportion of immediate family, extent of task sharing) and psychosocial (e.g., network mean and variability in caregiver stress, family disagreement surrounding care) components. Chi-squares and ANOVAs test network differences in PLWD demographic characteristics. A four-profile network was found to have best fit based on the AIC and BIC: 1) Adjusted specialists (38% of sample)- immediate family network, limited task sharing, high psychosocial well-being; 2) Disagreeing (30%)- diverse network, sharing self-care tasks, mixed reports of overload and family disagreement; 3) Strained (16%)- diverse network, limited sharing, poor psychosocial adjustment; and 4) Generalists (16%)- immediate family generalists, extensive task sharing, caregiving burden. PLWDs’ race varied significantly by network profile (χ2=29.8, p<.001). In particular, 78% of the Strained networks were assisting White PLWD while 49% of the Generalist networks were assisting Latinx/other race PLWD. Networks varied psychosocially even when composition and task sharing were similar, suggesting the need to consider both factors in defining networks. How networks function may have critical implications for care quality and well-being of both caregivers and PLWD.

